# Dual-RNAseq Analysis Unravels Virus-Host Interactions of MetSV and *Methanosarcina mazei*

**DOI:** 10.3390/v14112585

**Published:** 2022-11-21

**Authors:** Finn O. Gehlert, Till Sauerwein, Katrin Weidenbach, Urska Repnik, Daniela Hallack, Konrad U. Förstner, Ruth A. Schmitz

**Affiliations:** 1 Institute for General Microbiology, Christian Albrechts University, 24118 Kiel, Germany; 2 ZB MED, Information Centre for Life Sciences, 50931 Cologne, Germany; 3 Central Microscopy, Christian Albrechts University, 24118 Kiel, Germany

**Keywords:** virus–host interaction, archaea, *Methanosarcina*, MetSV, dual-RNAseq, replication, lysis

## Abstract

*Methanosarcina* spherical virus (MetSV), infecting *Methanosarcina* species, encodes 22 genes, but their role in the infection process in combination with host genes has remained unknown. To study the infection process in detail, infected and uninfected *M. mazei* cultures were compared using dual-RNAseq, qRT-PCRs, and transmission electron microscopy (TEM). The transcriptome analysis strongly indicates a combined role of virus and host genes in replication, virus assembly, and lysis. Thereby, 285 host and virus genes were significantly regulated. Within these 285 regulated genes, a network of the viral polymerase, *MetSVORF6*, *MetSVORF5*, *MetSVORF2*, and the host genes encoding NrdD, NrdG, a CDC48 family protein, and a SSB protein with a role in viral replication was postulated. Ultrastructural analysis at 180 min p.i. revealed many infected cells with virus particles randomly scattered throughout the cytoplasm or attached at the cell surface, and membrane fragments indicating cell lysis. Dual-RNAseq and qRT-PCR analyses suggested a multifactorial lysis reaction in potential connection to the regulation of a cysteine proteinase, a pirin-like protein and a HicB-solo protein. Our study’s results led to the first preliminary infection model of MetSV infecting *M. mazei*, summarizing the key infection steps as follows: replication, assembly, and host cell lysis.

## 1. Introduction

Although our knowledge on viruses and phages, which are ubiquitous distributed in all habitats and over all kingdoms, is growing every day, the kingdom of archaea is underweight in the number of characterized viruses [1,2,3]. Most archaea are highly specific for their environment and, consequently, their metabolic pathways are unique for condensed groups and consortia. Based on this high level of specificity of the hosts, the diversity of respective viruses is predicted to be even higher [1]. More than 17 different groups of archaea viruses have been described to date, with more than 100 identified viruses, which is, compared to bacterial phages and the archaeal diversity, still a very low number [1,2,3]. Phages and viruses are characterized by many lifecycles and infection mechanisms due to their high specialization [1,2]. Lytic virus infections are commonly divided into five infection steps, namely (i) attachment and entry, (ii) disassembly and localization, (iii) genome replication, (iv) virion assembly and genome packaging, and (v) maturation and release [2]. For the attachment of the virus envelope to its host, cell interactions on protein level between virus envelope and cell surface structures are fundamental [2,4]. In the case of archaea, the knowledge on detailed mechanisms of attachment and DNA transfer into the host is scarce. One example is the entrance mechanism of the *Sulfolobus islandicus* rod-shaped virus 2 (SIRV2) into *Sulfolobus islandicus* LAL14/1. The entrance of SIRV2 was shown to be mediated by the interaction of the capsomere with pilus-like filaments of the host cell [5]. For the next step, the introduction of the viral genome, different mechanisms have been reported [2]. Once inside the cytoplasm, a lytic virus takes control of host cell processes to replicate and produce viral offspring. Different studies demonstrated that not only transcription translation machinery of the host can be used for the life cycle of a virus, but also that the host cell protein folding machinery is used for virus assembly [6,7,8,9]. In the final and lethal step of the lytic infection, cells are lysed and virus particles are released. This final egression process can be mediated via different mechanisms and is, again, highly understudied in archaea. In *Sulfolobus* systems, the important role of virus-induced surface structures were reported. Formation of pyramidal surface structures was shown to be induced by *Sulfolobus* viruses, namely *Sulfolobus* turreted icosahedral virus (STIV) and SIRV2. Viral offspring is released through these surface structures. [10,11,12].

In recent years, a handful of new viruses have been described in infecting methanoarchaea e.g., Drs3, Blf4, and the *Methanosarcina* spherical virus (MetSV), but their infection processes are mostly unknown [1,13,14,15]. As one example, MetSV has a narrow host range, infecting *M. mazei*, *M. barkeri*, and *M. soligelidi* growing as single cells [15]. The lytic virus was characterized as a member of the *Tectiviridae*, because of its morphology with the internal lipid membrane and a linear dsDNA genome approximately 10.5 kb in size, encoding 22 open reading frames [15,16]. The MetSV virus is the only archaeal virus within the *Tectiviridae*, whereas the other 76 members are targeting bacteria, for example, the extensively studied phage PRD1. The MetSV virus can lyse liquid host cultures of OD_600_ ≈ 0.2 within 4 to 5 h. Interestingly, no CRISPR-Cas-mediated defense reaction or adaptation was observed so far, which might be due to the very low activity of the present type IB and IIIC system or the rapid lytic process [15,17]. 

In this study we used a genome-wide transcriptome approach based on RNAseq data and quantitative reverse transcriptase (qRT)-PCR analysis to shed light on the molecular mechanisms of MetSV infecting *M. mazei* Gö1 (DSM 3647). 

## 2. Materials and Methods

### 2.1. Growth of M. mazei, MetSV Infection and RNA Preparation

Growth experiments and RNA preparation were performed based on six cultures of *M. mazei* DSM3647, which were grown in 50 mL minimal media with 150 mM methanol as carbon and an energy source described elsewhere [15,18,19]. Four cultures were infected with MetSV by adding 500 µL of previously virus lysed *M. mazei* DSM3647 culture when mean optical density measured at 600 nm (OD_600_) reached ≈ 0.2. Two uninfected cultures were used as control samples. Cultures were harvested by centrifugation (1756× *g*; 4 °C; 20–30 min), at 30 or 180 min post-infection (p.i.). Pellets were resuspended in 1 mL Roti-Zol (Carl Roth GmbH, Karlsruhe, Germany), mixed with 200 µL chloroform and centrifuged at 12,000× *g* and 4 °C for 3 min. The RNA-containing supernatant was transferred and RNA was precipitated using 500 µL 2-propanol followed by 10 min incubation at room temperature (RT) and the same centrifugation protocol. Pellets were washed once with 500 µL ice-cold ethanol (70%) by centrifugation (12,000× *g*; 4 °C; 5 min) and dissolved in 30 µL RNase-free water after drying at RT. The RNA samples were treated twice with DNase1 (Invitrogen, Waltham, MA, USA) following the manufacturer’s protocol and tested for remaining DNA by PCR with primers targeting *MM_RS08385* [*MM_1621*] and *MetSVORF9* followed by agarose gel electrophoresis (Table S1).

### 2.2. RNA Sequencing

Processing, cDNA synthesis, library preparation, and sequencing were performed by the core unit system medicine at the University of Würzburg. The RNA quality was checked using a 2100 Bioanalyzer with the RNA 6000 Nano kit (Agilent Technologies, Santa Clara, CA, USA). Here, cDNA libraries suitable for sequencing were prepared from 500 ng of total RNA, fragmented for 2 min and 45 s at 94 °C and treated with T4 PNK for phosphorylation/dephosphorylation and RppH for decapping, followed by NEBNext^®^ Multiplex Small RNA Library Prep. (New England Biolabs, Ipswich, MA, USA) without rRNA depletion. The number of the PCR cycles was determined as 13 by qPCR and the elongation time was set to 30 s. Libraries were quantified by a Qubit^TM^ dsDNA HS Assay Kit 3.0 fluometer (ThermoFisher, Waltham, MA, USA) and quality was checked using a 2100 Bioanalyzer with a high-sensitivity DNA kit (Agilent Technologies) before pooling. Sequencing of pooled libraries, spiked with 5% PhiX control library, was performed in single-end mode with 150 nt read length on the NextSeq 500 platform (Illumina, San Diego, CA, USA) with a Mid Output Kit (Illumina).

### 2.3. Read Trimming, Filtering and Mapping Using READemption

Illumina reads were trimmed prior to read mapping using cutadapt (version: 1.16) [20]. Illumina’s TruSeq “Read 1” adapter sequence was removed from the 3′ end. Additionally, nucleotides with a Phred quality score lower than 20 and their following downstream (5′ to 3′) bases were cut off. Further filtering steps, read mapping, and downstream analysis, i.e., gene quantification, generation of coverage files and differential gene expression analysis were carried out by the RNA-seq tool READemption (version: 0.4.3, https://doi.org/10.5281/zenodo.250598) [21]. Further read filtering included clipping of poly(A) sequences and discarding of reads that had a read length below 20 nucleotides after performing the trimming steps. The short read mapper segemehl (version: 0.2.0) [22], which is integrated into READemption, was used for read mapping. The mapping was performed with a mapping accuracy of 90% and segemehl’s realigner lack [23]. The archaeal genome and annotation were obtained from NCBI’s RefSeq database (accession number: NC_003901.1, RefSeq assembly accession number: GCF_000007065.1), while the viral ones were obtained from NCBI’s genbank (accession number: MF186604.1, Genbank assembly accession number: GCA_002990055.1). The archaeal annotation was extended with sRNA and mass spectrometry data generated in previous studies [24,25]. The gene quantification files i.e., the number of reads overlapping with an annotated feature and the coverage files in wiggle format, i.e., the number of reads overlapping with each base of the genome, were created using READemption (version 1.0.5). Afterwards, both file types were split up by species. The coverage was normalized by the total number of aligned reads of a given replicate and multiplied by 1,000,000. The gene quantification counts were normalized by the transcripts per million method [26]. 

### 2.4. Differential Expression Analysis by Clusters

Differential expression analysis was realized with the R package “DESeq2” (version 1.34.0, R version 4.1.3 (10 March 2022)) based on raw read count [27]. The dataset was not split by species for model generation, because MetSV is not able to express genes on its own, in comparison to bacterial–eukaryotic systems. Genes with an adjusted (Benjamini–Hochberg-corrected) *p*-value equal to or less than 0.05 and a Log2FoldChange (Log2FC) value < −2.5 or >2.5 were defined as differentially transcribed. Visualization of a regulation pattern was performed in R using log-transformed tpm data by circular heatmaps using the “circlize” package (version 0.4.14; [28]) and “ComplexHeatmap” (version 2.10.0; [29,30]). For this purpose, tpm values were rounded and zero values were replaced by 1 before data was log-transformed to circumvent NAs and finally clustered by regulation using the R package “stats” (functions dist and hclust; [31]). Visual output was used to further subset the data to subtrees, which were than further analyzed and visualized using “ggplot2” (version: 3.3.5; [32]) and GraphPad Prism (GraphPad Prism version 9.3.1 for Mac, GraphPad Software, San Diego, CA, USA). Coding sequences of *M. mazei* and MetSV were categorized by using eggNOG-mapper-2.1.7 (eggNOG) [33,34] working with diamond version 2.0.14 [35] on a ×86 Linux cluster. Clusters of orthologous gene (COG) categories were matched to READemption and DESeq2 output.

### 2.5. Verification by qRT-PCR

Here, qRT-PCR was used to verify RNAseq results. For viral transcripts, a plasmid-based normalization method was established to decouple virus and host transcription by not using host-derived housekeeping genes (absolute qRT-PCRs). For this purpose, *MetSVORF15*, encoding the small capsid protein, was PCR-amplified from viral DNA using 5-CATATGGTCGACTTAGTACC-3 and 5-GAATTCTTACCAATTGTCGATG-3, digested with NdeI and EcoRI (New England Biolabs GmbH, Frankfurt am Main, Germany) and ligated to pET28a (+) (Novagene^®^, Merck Millipore, Darmstadt, Germany), resulting in *MetSVORF15* plasmid pRS1332. This pRS1332 was isolated, and its concentration was determined using the nanodrop technique. For qRT-PCR, a serial 10-times dilution of pRS1332 with 2 technical replicates for each dilution was used and set up with the same qRT-PCR Kit (QuantiTect SYBR Green RT-PCR Kit, Qiagen, Hilden, Germany). The qRT-PCR reactions were performed based on [36,37]. Calculated total DNA amounts of the serial dilutions were used to calculate the total molecule number, using the following Equation (1), as based on [38]:

(1) $${\mathrm{transcripts}}_{\mathrm{total}}=\frac{6.022*10^{23}\left(\frac{\mathrm{transcript}}{\mathrm{mol}}\right)*\mathrm{DNA}\;\mathrm{amount}\left(g\right)}{\mathrm{DNA}\;\mathrm{length}\left(\mathrm{bp}\right)*660\;\left(\frac{g*\mathrm{mol}}{\mathrm{bp}}\right)}$$

Calculated molecule numbers on a logarithmic scale were plotted against the measured ct-values. Total number of transcripts were calculated by the exponential trendline and the measured ct-values for the different open reading frames. For *M. mazei* transcripts, qRT-PCRs with normalization based on housekeeping genes were used (relative qRT-PCRs), as described in [36,37]. FoldChange was transformed to Log2FoldChange using the “base” R package [31].

### 2.6. Ultrastructural Analysis by Transmission Electron Microscopy (TEM)

After 30 or 180 min post-infection (p.i.), cultures were fixed by adding glutaraldehyde to a final concentration of 1% and then incubated for 10 min at 37 °C followed by 2 h at RT. Cells were harvested by centrifugation for 10 min at 1000× *g* and 4 °C and were resuspended in 500 µL fresh fixative buffer (100 mM Hepes, pH 7.0, 1% glutaraldehyde). Cells were incubated overnight at RT and then stored at 4 °C. For Epon resin embedding, cells were embedded in 1% low melting point agarose, post-fixed with 1% OsO_4_ in 1.5% potassium ferricyanide for 1 h on ice, contrasted with 2% aqueous uranyl acetate en bloc for 2 h, dehydrated with a graded ethanol series (70-80-90-96-100%), and progressively infiltrated with Epon resin. Heat-polymerized blocks were sectioned in a Leica UC7 ultramicrotome. Thin 80 nm sections were deposited on copper, slot, formvar-coated grids, and contrasted with saturated aqueous uranyl acetate for 10 min, followed by 0.03% lead nitrate for 3 min. Grids were imaged in a CM10 transmission electron microscope (Philips), operated at 80 kV, and equipped with a LaB6 filament, a CCD side-mounted MegaView III camera, and iTEM software (both from Olympus Soft Imaging Solutions).

## 3. Results

### 3.1. TEM Imaging of MetSV-Infected M. mazei Cells

**Figure 1:**
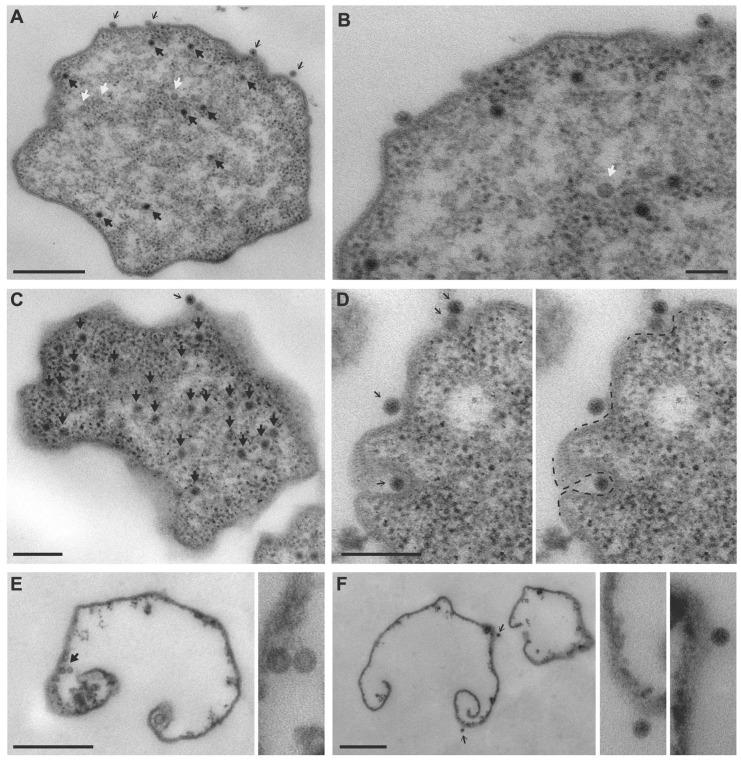
Ultrastructural analysis of MetSV-infected *M. mazei* cells at 180 min p.i. (**A**) Infected cell with MetSV particles randomly scattered through the cytoplasm (block arrows) or attached along the cell surface (arrows). White arrows point to less electron-dense virus particles, possibly representing particles with incomplete DNA packaging. (**B**) Part of the same cell imaged at higher resolution. The S-layer and cell membrane can be resolved at the surface of the cell. (**C**) Infected cell with a higher viral load. (**D**) Occasionally, surface-attached viral particles were observed in membrane pockets (dashed line). (**E**,**F**) Membrane remains representing lysed cells as strongly suggested by virus particles associated with the membrane either at the cytoplasmic (block arrow) or the extracellular side (arrow). Enlarged particles to the right of the main images. Scale bar, (**A**) 1 μm, (**B**) 100 nm, (**C**,**D**) 200 nm, and (**E**,**F**) 500 nm.

Ultrastructural analysis was performed to visualize the interaction between MetSV particles and *M. mazei* cells. At 30 min p.i., *M. mazei* cells showed no obvious signs of infection and no MetSV virus particles could be found on the thin sections. Even when a culture was infected at a 100-fold higher virus titer, only a few cells with single virus particles attached to the cell surface were observed (Figure S1). At 180 p.i., the infection became obvious (Figure 1). There were many cells with multiple MetSV particles scattered throughout the cytoplasm, and some cells also had virus particles attached to the surface. Virus particles inside cells appeared randomly distributed, and no clustering within the cytoplasm or at the cell membrane could be observed (Figure 1A–C). A few less electron-dense MetSV particles could be detected, potentially with incomplete DNA packaging (Figure 1A,B), but most of the particles had an electron-dense core, indicating nucleic acid (DNA) content. The attachment site of MetSV did not show preference for any specific surface structure e.g., filaments or pili. In rare cases, viral particles were found in membrane pockets with different depths (Figure 1D). The membranes of most infected cells appeared intact. However, a few cases of membrane remains associated with ribosomes at the cytoplasmic side and often with few virus particles attached at either side indicated that some infected cells had already been lysed at 180 min p.i. (Figure 1E,F). It is probable that virus particles attached to the surface of cells a t180 min p.i. had been released from lysed infected cells in the culture and had, thus, started a new life cycle.

### 3.2. Dual-RNAseq Analysis–READemption, DESeq2 and eggNOG Results

#### 3.2.1. MetSV Is Globally Changing *M. mazei* Transcriptome

**Figure 2:**
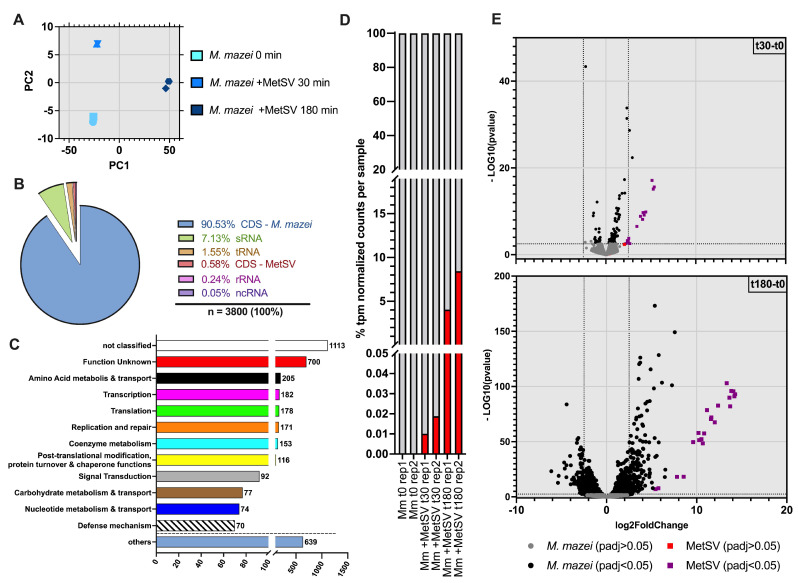
Summary of dual-RNAseq analysis of MetSV-infected *Methanosarcina mazei* cells. Total RNA of MetSV-infected *M. mazei* cultures was isolated at defined time points post-infection (p.i) (t0, uninfected; t30 and t180, 30 and 180 min p.i., respectively), sequenced, and analyzed based on READemption (version 1.0.5), DESeq2 (version 1.34.0) and eggNOG-mapper-2.1.7 output. READemption output was normalized by the transcript per million method (tpm). Here, DESeq2 was used for analysis of differentially transcribed genes in comparisons between conditions (t30 vs. t0 and t180 vs. t0). (**A**) Principal component analysis (PCA) showed similarity of two biological replicates and differences in time points based on the DESeq2 model. Here, light blue is untreated cells; blue is virus-treated cells at 30 min p.i.; dark blue is virus-treated cells 180 min p.i. (**B**) Dataset contained, in total, 3800 genes (3778 *M. mazei* ORFs and 22 MetSV ORFs), which were categorized into the following groups of RNA types based on their annotations: CDS/ORFs (*M. mazei* or MetSV), sRNAs, tRNAs, rRNAs, and not further classified ncRNAs (*M. mazei*). (**C**) Overview about abundances of defined clusters of orthologous genes (COG) categories of *M. mazei* and MetSV genes within the dataset based on eggNOG-mapper-2.1.7 and NCBI database results (Database download on 04/2022). Categories “Chromatin & Dynamics”, “Energy production & conversation”, “Cell cycle control & cell division”, “Cell wall, membrane & envelope biogenesis”, “Lipid metabolism”, “Secondary structure”, “Cell motility”, “Intracellular trafficking, secretion & vesicular transport”, and “inorganic ion transport & metabolism” were grouped in “others”. (**D**) Changes in the percentages of transcript per million (tpm) normalized read counts for virus (red) and host (gray) over time per analyzed sample. (**E**) Volcano plots showing regulation of transcripts in the comparisons t30 vs. t0 and t180 vs. t0. The *y*-axes represent log10-transformed *p*-values and the *x*-axis shows Log2FoldChanges (Log2FC). Significance threshold *p*-value = 0.05; thresholds for regulation (Log2FC) is <−2.5 and >2.5. For *M. mazei* genes, gray is not significant, and black is significant; for MetSV genes, red is not significant, and purple is significant.

The global transcriptome response of *M. mazei* to MetSV infection was evaluated using a dual-RNAseq approach. Two time points during the infection, namely 30 and 180 min post-infection (p.i.), were investigated in comparison to uninfected cells (0 min p.i.) with two biological replicates each. The obtained dataset was analyzed by the “DESeq2” and “stats” package in R for differentially transcribed virus and host genes as described in the methods of [27,31]. Principal component analysis of the DESeq2 model showed a close similarity between replicates but expected variation between conditions (0 min, 30 min, and 180 min) (Figure 2A). This similarity was observed, as well as taking a closer look at the transcript per million (tpm) normalized read counts per replicate (Figure 2D). The percentage of viral reads increased over time in comparison to the host reads (Figure 2D). A strong increase in viral transcripts was observed between 30 min and 180 min p.i. (mean values of biological replicates were as follows: 0.014 ± 0.006% to 6.246 ± 3.112%; Figure 2D). A total of 99.42% of the 3800 detected genes of the current dataset were annotated on the *M. mazei* genome, whereas 0.58% belonged to MetSV. The majority of *M. mazei* genes (90.53%) were coding sequences (CDS) in prokaryotes equivalent to open reading frames (ORFs), while MetSV genes were exclusively CDS (Figure 2B). Furthermore, *M. mazei* RNA species showed 7.13% sRNA, 1.47% tRNA, 0.24% rRNA, and 0.05% not further classified ncRNA (Figure 2B) (Table S2). The number of significantly regulated genes (Log2FoldChange of <−2.5 and >2.5) increased between 30 min and 180 min p.i. by a factor of more than 21 (Figure 2E). Two coding genes of *M. mazei*–*MM_RS14840* and *MM_RS16695*–and 50% of the virus genes were differentially transcribed at 30 min p.i. (Figure 2E). At 180 min p.i., in comparison to untreated cells, the number of significant differentially transcribed *M. mazei* ORFs strongly increased up to 261, with 80 genes less transcribed and 181 more transcribed (Figure 2E). At this time point, all 22 MetSV ORFs were upregulated significantly (Figure 2E). To obtain information about pathways and processes influenced by viral infection, all host and virus genes annotated on their genomes were analyzed by eggNOG-mapper-2.1.7 for the main categories (Figure 2C) ([34]; Database download: 04/2022) (Table S2). The analysis showed that all categories, except “RNA processing and modification”, “Nuclear_structure”, “Cytoskeleton”, and “General_Functional_Prediction_only” were present in the transcriptome dataset. The groups of “Defense mechanism” (*n* = 70), “Replication and repair” (*n* = 171), and “Post-translational modification, protein turnover, chaperone functions” (*n* = 116) groups were especially in our focus, due to expected differences in transcription during MetSV infection. Additionally, a high number of ORFs were grouped in the “unknown function” (*M. mazei*, 699; MetSV, 1) group or as “not classified” (*M. mazei*, 752; MetSV, 20) (Table S2).

#### 3.2.2. Transcription of Viral Replication Related Genes

**Figure 3:**
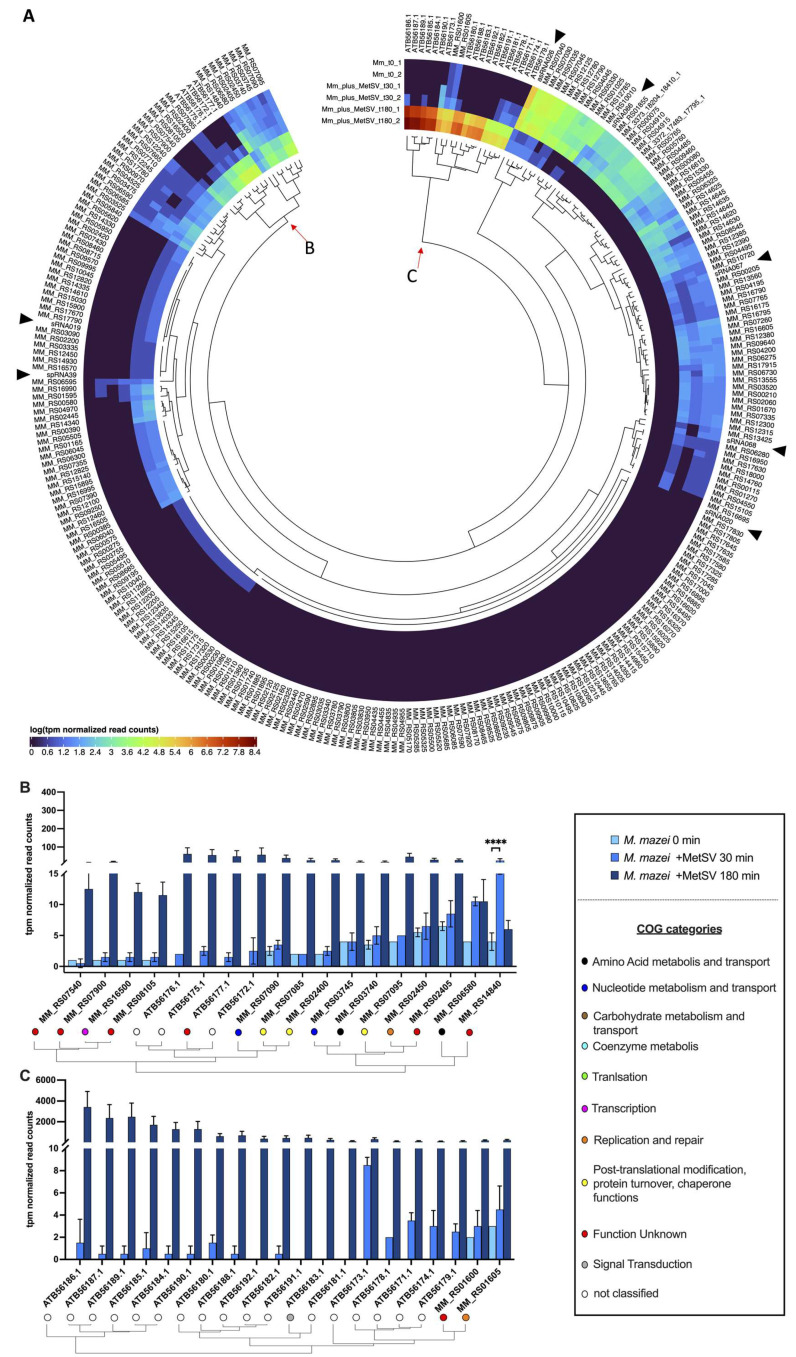
MetSV infection induced significant changes to the *M. mazei* transcriptome. (**A**) Selection of significant regulated transcripts (DESeq2 results, with a *p*-value = 0.05; thresholds for regulation (Log2FC) of <−2.5 and >2.5) for comparisons of t30-t0 (2 genes) and t180-t0 (285 genes) visualized in a circular heatmap based on log-transformed transcript per million (tpm) normalized read counts using the R package “circlize” (version 0.4.14) and clustered by regulation using the “stats” (version 4.2.0) package (functions were hclust() and dist()). Black arrows are highlighting significantly regulated sRNAs. (**B**) and (**C**) are zooming into nodes and show clustered bar plots of tpm-normalized read counts (*y*-axis) and gene IDs (*x*-axis). Clustering was performed as described in (**A**), and was supplemented with clusters of orthologous genes (COG) categories (EggNOG-mapper version 2.1.7 and NCBI database). All represented genes (285) were significantly regulated in the comparisons between t180-t0, and there was only one significant differentially transcribed gene in the comparison between t30-t0, as indicated by stars (two-way ANOVA; *p*-values were ≤0. 0001 = ****).

When searching for relevant transcript regulations, a subset of significant regulated genes (<0.05 *p*-value (adjusted); Log2FC < −2.5 or >2.5) was generated and aligned to the eggNOG results (Table S3). The final subset contained 285 genes out of, initially, 3800 (*M. mazei* and MetSV) (Figure 3). Genes were clustered based on a log-transformed regulation pattern of tpm nomalized read counts. The focus of the analysis was on branches where host and viral genes clustered together. Two branches at the ends of the circular heatmap were identified. The first cluster showed four viral protein coding genes, namely ATB56176.1 (MetSVORF6), ATB56175.1 (MetSVORF5), ATB56177.1 (MetSVORF7, DNA polymerase of Type B), and ATB56172.1 (MetSVORF2), and *M. mazei* genes out of the following different COG categories: “transcription”, “nucleotide metabolism and transport”, “post-translational modification, protein turnover, chaperone functions”, “amino acid metabolism and transport”, “replication and repair”, and “function unknown” (Figure 3B). Within one sub-branch, the four viral genes, and especially the viral polymerase, were clustering with *MM_RS07090* (NrdD), *MM_RS07085* (NrdG), and *MM_RS02400* (CDC48 family protein). The *MM_RS07095* (NrdH) was found in a closely associated cluster together with the gene *MM_RS14840*, encoding a protein with an unknown function. All described genes showed the same trend, namely low expression values in the beginning and increasing over time, except *MM_RS14840*, which had its transcription maximum at 30 min p.i. (Figure 3B). The second cluster showed two *M. mazei* ORFs, namely *MM_RS01600* and *MM_RS01605* (RepA_1_), and all the remaining virus genes (Figure 3C). The *MM_RS01605* codes for the single strand DNA (ssDNA) binding (SSB) protein RepA_1_, which has a potential role in viral replication, based on its oligonucleotide binding (OB)-fold motif.

#### 3.2.3. Effects of MetSV Infection on *M. mazei*-Derived Defense Genes

Although no genes of the “Defense mechanism” category could be detected in these two selected clusters (Figure 3), five potential defense genes were found in the whole set of significant differentially transcribed genes. Two of those genes, *MM_RS00575* and *MM_RS16605*, were annotated as multi-antimicrobial extrusion (MatE) proteins, while *MM_RS01080* and *MM_RS028955* code for a type III restriction enzyme and a not characterized putative restriction endonuclease subunit S [*MM_RS02895*]. Furthermore, *MM_RS16885*’s so-called *cas1solo* was significant differentially transcribed in virus-infected cells. The *MM_RS16885* was previously described to be a solitary Cas1 family protein not in the context of CRISPR loci and CRISPR immunity [17,39], but rather as a proposed transposase of a new class of transposons (casposons) [40]. In the context of the current analysis, differential transcription under MetSV influence on the two reported CRISPR loci was investigated; however, no differences in transcription in comparison to uninfected cells could be detected.

#### 3.2.4. Transcriptional Changes of Further Genes and Gene Categories

Further significant differentially transcribed genes with potential meaning for the virus infection were detected. The transcript levels of *MM_RS01165* (cysteine proteinase) increased between 30 min and 180 min p.i. (>25 fold), the same increase was obtained for *MM_RS15030* (pirin family protein; >15 fold) and for *MM_RS17670* coding for a solitary HicB protein. In type II toxin-–antitoxin systems, HicB antitoxins are usually present with a corresponding HicA toxin. Beside those solitary genes, the MetSV infection had a global impact on the transcription of amino acid metabolism-associated operons. The strongly regulated tryptophan operon (trp) [*MM_RS14615*-*MM_RS14645*] encoding all necessary enzymes for tryptophan biosynthesis was, to some extent, upregulated at 30 min p.i. (>1.3–4.6 fold), followed by a strong decrease 180 min p.i.

#### 3.2.5. Infection Changes of Host sRNAs Transcription

**Figure 4:**
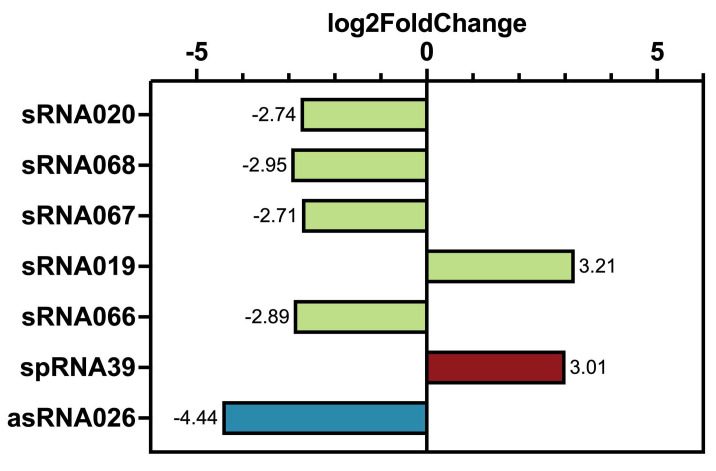
Impact of MetSV infection on transcription of ncRNAs, spRNAs, and asRNAs. Significant regulated sRNAs (comparison t180 vs. t0) are summarized (DESeq2 results with a *p*-value = 0.05; thresholds for regulation (Log2FC) are <−2.5 and >2.5). The sRNAs were not significantly regulated in a comparison of t30 vs. t0. Colors represent different types of sRNAs, as follows: not further classified sRNAs (light green), small protein encoding sRNAs (spRNAs, red color) and antisense RNAs (asRNAs, blue color).

Within the subset, host-derived sRNAs were also detected. Five ncRNAs, one spRNA, and one asRNA were found to be significant differentially transcribed, but these did not belong to one of the previously described clusters (Figure 3A and Figure 4). Transcription levels of sRNA019 and spRNA39 increased over time, whereas all the other sRNAs of the subset were less transcribed in infected cells (t180 vs. t0) (Figure 4). Here, sRNA019 was identified in *M. mazei* growing under nitrogen stress conditions; however, no target has been identified so far [24]. Thus, it is tempting to speculate that the respective gene products of their mRNA targets are involved in virus propagation or cell lysis, whereas the down-regulation of the other sRNAs might be involved in decreasing the metabolism of *M. mazei*.

#### 3.2.6. Classification of Virus Genes and Verification of RNAseq Results by qRT-PCR

**Figure 5:**
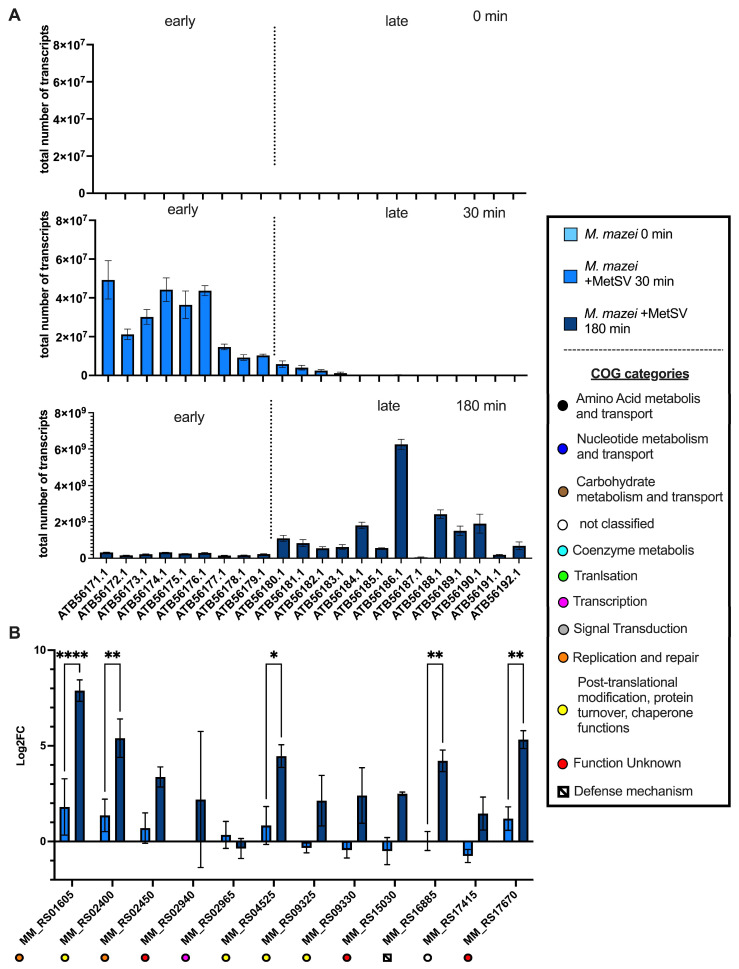
qRT-PCR analysis verifying the transcript regulation of host and virus genes. (**A**) Absolute transcript numbers based on qRT-PCR analysis of virus ORFs separated by time point and normalized to serial dilution of the pRS1332 plasmid. (**B**) Log2FCs of the comparisons t30 vs. t0 and 180 vs. t0 of selected *M. mazei* genes are depicted, and corresponding clusters of orthologous gene (COG) categories can be found in the lower panel (EggNOG-mapper version 2.1.7 and NCBI database). Significances are as follows: based on ttwo-way ANOVA (*p*-values are ≤ 0.05 = *, ≤ 0.01 = **, and ≤ 0. 0001 = ****).

A subset of identified virus and host genes were verified with absolute and relative qRT-PCR as described in the Methods section. In general, qRT-PCRs showed equal results to the dual-RNAseq analysis (Figure 5). Viral transcription analysis was further used to categorize viral ORFs into “early” and “late” genes (Figure 5A). Due to the faster increase in the transcription levels of ATB56171.1-ATB56179.1 (MetSVORF1-MetSVORF9), which were more abundant than ATB56180.1-ATB56192.1 (MetSVORF10-MetSVORF22) at 30 min p.i., these ORFs were marked as “early genes”, whereas all the other ORFs were marked as “late genes”. No “intermediate” genes were detected. Relative qRT-PCRs of host genes verified the strong significant (two-way ANOVA, Šídák’s corrected for multiple comparisons) increase in *MM_RS01605* (RepA_1_) during the virus infection (*p* < 0.0001; Figure 5B). Further genes were equally significant regulated e.g., *MM_RS02400* (CDC48 family protein, p_adj_ = 0.0093), *MM_RS04525* (Signal peptide peptidase A (SppA), p_adj_ = 0.0247), *MM_RS17670* (HicB family antitoxin, p_adj_ = 0.0076), and *MM_RS16885* (Cas1solo, p_adj_ = 0.0065). Here, GroES and and GroEL, two subunits of a chaperone complex, encoded by the genes *MM_RS09325* and *MM_RS09330*, were transcribed in higher amounts a t180 min p.i., but this increase was not significant in comparison to the uninfected cells (Figure 5B).

## 4. Discussion

### 4.1. Transmission Electron Microscopy Demonstrates Key Steps in MetSV Infection

The MetSV particles attached to *M. mazei* cells were rarely observed at 30 min p.i., even if a 100-fold higher virus titer was used. This could be explained by the lower probability in detecting rare events on thin sections compared to observations of whole cells. At 180 min p.i., some infected cells had already been lysed, and they had released many copies of virus particles into the medium. Therefore, the viral load in a culture increased considerably and virus particles attached to the *M. mazei* cell surface could readily be observed. These virus particles seemed randomly distributed along the cell surface and did not show any preferences for specific surface structures e.g., flagella, as was described for at least some viruses and phages as reviewed in [4]. The attachment of PBS1 to the *Bacillus subtilis* flagellum can serve as one early example [41]. One example found in archaea is the attachment of the *Sulfolobus* virus SIRV2. In this case, SIRV2 was shown to interact with the ends of thin (5–10 nm diameter) filaments [5]. In contrast to these findings, MetSV particles were attached either directly to the surface-layer (S-layer) proteins of *M. mazei* themselves or to S-layer-associated proteins. However, thin sections are not suitable for the visualization of tiny filaments and the interaction between the filaments and MetSV can be excluded only if whole archaea cells are imaged (for example by negative staining). A comparable interaction of MetSV and glycosylated S-layer proteins, as described for the virus ϕCh1 infecting *Natrialba magadii*, can be predicted [42]. Furthermore, TEM imaging suggested randomized MetSV particle production in the host cells. Although the clustering of archaeal viruses prior to cell lysis could be shown for the *Sulfolobus* virus STIV [11,12], this was not observed for MetSV particles, which were found to be un-clustered in infected *M. mazei* cells. Although MetSV contains an internal lipid membrane with the same lipid composition as the host cell membrane, viral assembly was not associated with the cell membrane [15]. This suggests the de novo synthesis of viral lipid membrane.

In previous reports, specific archaeal surface structures in the context of host cell lysis and viral particle release were described. These structures were described for *Sulfolobus* viruses STIV and SIRV2 [10,11,12]. However, comparable surface structures were not found in MetSV-infected *M. mazei* cells. Based on the membrane remains found at 180 min p.i., we propose that infected cells burst at single and non-specific sites, probably by general membrane destabilization. The latter could be a consequence of a systemic lysis reaction, whereby the host gene expression in combination with viral proteins leads to an unspecific destruction of the host cell. Dual-RNAseq and qRT-PCR were next used to gain a deeper understanding of the lysis mechanism and underlying gene regulation, as well as to unravel MetSV replication and assembly.

### 4.2. Dual-RNAseq

The number of publications based on dual-RNAseq techniques has been increasing since 2012. More than 150 dual-RNAseq studies were accessible on Pubmed in the first half of 2022. Those studies focused on different cell types and pathogens. Eight publications were investigating virus–host interactions, focusing on eukaryotic organisms infected with different types of viruses e.g., SARS-CoV-2, herpes viruses, or influenza viruses, but to our knowledge this report on *M. mazei*/MetSV is the first to characterize an archaeon–virus system with a dual-RNAseq approach [43,44,45]. In comparison to the majority of dual-RNAseq studies, virus–host systems have one major issue, due to the fact that viruses or phages do not have a (complete) transcription–translation machinery on their own. Thus, host and aggressor gene expressions are inseparable, making it crucial to perform DESeq2 analysis with a combined dataset of virus and host ORFs, as was recently reported by Maulding and colleagues in 2022 with human cells infected by SARS-CoV-2 [43]. For this reason, the final dataset of the current study combined 285 significant differentially transcribed genes out of the 3800 detected *M. mazei* and MetSV genes in comparisons of the investigated time points (0 min p.i., 30 min p.i. and 180 min p.i.). This subset was than further analyzed to allow the first insights in the molecular mechanism of infection. First, the focus of the current analysis was given to host defense reactions, e.g., CRISPR-related defense and additional potential defense genes through classification by eggNOG.

#### 4.2.1. *M. mazei* Defense Reactions to Prevent MetSV Infection

Defense reactions of *M. mazei* within known defense modules were investigated, but no different transcription was observed. The two reported CRISPR loci of type IB [*MM_RS02925*-*MM_RS02965* and array (genome localization at 682,638-679,197)] and type IIIC [*MM_RS17410*-*MM_RS17445* + array (genome localization at 4,095,296-4,089,310)] [17] were not significantly differentially transcribed. The lack of a detectable Cas transcription of the previously presented genome loci agreed to early findings of only weak CRISPR Cas activity in *M. mazei* [17]. Only one gene belonging to the Cas1family was found within the dataset to be upregulated due to the virus challenge, but this encodes the so-called Cas1solo. Cas1solo was described as the key enzyme of a casposon, belonging to new group of DNA transposons. The enzyme was not found to replace or complement a potentially not functional Cas1 enzyme of the CRISPR system, so far [39,40,46] (Figure 3 and Figure 4). The observed increase in transcription was in a low range based on RNAseq results and on relative qRT-PCRs, and might be a hint for general stress-induced transposase activity, which was described for a variety of organisms, stress conditions, and transposons [47,48,49,50]. For example temperature stress-induced translocation of transposons was reported in *Halobacterium halobium* [50]. Infections with different kinds of viruses were described to mediate increased transposon activity in a variety of human and mouse tissues [51]. Beside this, four more genes, categorized by eggNOG with roles in putative “defense mechanisms”, were significant differentially transcribed during a virus challenge. Two of these were encoding multi-antimicrobial extrusion (MatE) domain containing proteins (*MM_RS00575*; *MM_RS16605* (*dinF*)). The MatE proteins play a role in the excretion of toxic compounds, which could hypothetically include viral proteins or degradation products. Excretion of MetSV-encoded proteins or peptides could be one *M. mazei*-derived defense mechanism to prevent virus production in the cell. *DinF* was described as part of the SOS response and was discussed to reduce bile salt-derived reactive oxygen species (ROS) and ROS-mediated oxidative stress, especially for DNA, which was found in *Escherichia coli* [52,53]. The *M. mazei*-encoded *DinF* was less transcribed during MetSV infection, which would disagree with its role in defense, or it could be inhibited by MetSV-encoded genes or MetSV-induced pathways. The significant differences in transcription of the two genes *MM_RS01080* and *MM_RS02895* encoding restriction enzymes potentially belonging to the *M. mazei* restriction-modification system (RM). Both enzymes could putatively mediate cleavage of invading DNA, but this defense reaction is based on transcriptional levels and the rapid decline in cell densities in infected cultures too weak to inhibit MetSV lytic infection [54]. To further confirm this lack of a defense response, the *M. mazei* genome was analyzed using PADLOC version 1.1.0 [55] to search for novel defense modules or defense-related genes (Table S4). Within the subset of significantly regulated genes, only one gene (*MM_RS00580*) was found belonging to a defense module not further classified (“DMS_other”), encoding a potential transcription factor with an unknown target.

#### 4.2.2. Clusters of Co-Transcribed Genes of MetSV and *M. mazei* Reveal Interaction Network in Viral Replication

Differential expression analysis by clusters was performed to obtain a better understanding of co-transcribed genes and to hypothesize a potential role for their encoded proteins in interaction networks. For this analysis, the subset of significant differentially transcribed ORFs was clustered by regulations based on log-transformed read counts. Due to viral-induced host genome damage or degradation, which was described for SIRV2 infected *S. islandicus* LAL14/1 cells [56], the number of sequencing reads and the mean length of transcripts could be affected during the experiment, so the read counts were transcript per million (tpm) normalized, which made it possible to compare read counts of genes between time points, similar to work described in different publications [57,58,59].

The analysis led to the selection of two cluster sub-branches, which combined viral and host genes building up a potential interaction network. One cluster consisted of the viral polymerase MetSVORF7 [*ATB56177.1*], MetSVORF6 [*ATB56176.1]*, the small viral proteins MetSVORF5 [*ATB56175.1*], and MetSVORF2 [*ATB56172.1*], together with the *M. mazei* proteins anaerobic ribonucleoside–triphosphate reductase NrdD [*MM_RS07090*], anaerobic ribonucleoside–triphosphate reductase-activating protein NrdG [*MM_RS07085*], and the CDC48 family protein encoded by *MM_RS02400*. Although the role of this cluster is unknown so far, an interaction network on the protein level can be postulated in context of viral replication. Due to the genome architecture of MetSV as a double stranded DNA (dsDNA) virus with terminal inverted repeats (TIRs), a replication mechanism similar to other linear dsDNA viruses and phages e.g., adenoviruses, phi29, or PRD1 seems to be obvious [60,61,62]. Linear replication of phi29 is mediated by a complex formation of a terminal covalently bound protein (TP) and the DNA polymerase of type B. Additionally, replication is highly dependent on single strand DNA binding proteins (SSB), which are, in the case of phi29 phage-encoded and deoxynucleotide triphosphates (dNTPs), the crucial DNA building blocks [61]. The operon consisting of the genes *nrdH*, *nrdD*, and *nrdG* encode proteins, which are necessary for dNTP production from NTPs under anaerobic conditions and are, therefore, essential for DNA replication of MetSV or its host *M. mazei* [63]. The NrdG is necessary to activate NrdD, which catalyzes the reaction of NTPs to dNTPs in an oxygen-free environment as reviewed in [63], and might also have a role in oxidative stress resistance, as described for the *Corynebacterium glutamicum* homologue [64]. For MetSV, no SSB proteins were annotated so far, so the usage of host-encoded SSBs, such as RepA_1_ [*MM_RS01605*] and its operon with *MM_RS01600* and *MM_RS01595*, would close this gap. The RepA_1_ protein is necessary in MetSV linear replication to keep DNA single stranded and to protect it against nucleases in a similar way as the phage-encoded SSBs p5 in phi29 or P12 in PRD1 [65]. Genes encoding the small viral proteins MetSVORF2, MetSVORF5, and MetSVORF6 clustering together with *M. mazei* genes, as described above, were all transcribed in the early infection phase. Because of these facts, these proteins are postulated to play an important role in viral replication. Due to the lack of a known TP-encoded protein on the MetSV genome, there seems to be a high probability for a similar role of these viral proteins alone or in a heterocomplex, based on this cluster analysis. Furthermore, MetSVORF6, carrying a known DNA binding motif, is an attractive candidate, and might be essential for the replication process [15].

The increase in *MM_RS02400* (CDC48 family protein) transcription during MetSV infection could be explained by a strong and consistent increase in viral transcripts encoding proteins with putative toxic effects on host cell metabolism. This strong protein expression potentially leads to higher amounts of aggregated proteins in inclusion bodies [66]. The *MM_RS02400* coding for a CDC48 family protein has a potential function in disaggregation of inclusion bodies, as was described during the *Tobacco mosaic virus* (TMV) infection of plants [67]. Disaggregation of MetSV proteins could be beneficial for the viral infection cycle, due to higher levels of soluble viral proteins, or it could be a process to degenerate viral proteins by the host via 26S proteasome.

#### 4.2.3. Role of Host Genes in MetSV Particle Assembly

Viral assembly might be assisted by host chaperones e.g., *MM_RS09325* (GroES) and *MM_RS09330* (GroEL), which were the most prominent chaperones in the actual dataset, although these genes were not significantly differentially transcribed based on dual-RNAseq analysis and relative qRT-PCRs. The GroEL and GroES were found to play a crucial role in the assembly of a variety of phages e.g., lambda, T5 and PRD1 [8,9]. MetSV and PRD1 are both belonging to the *Tectiviridae*, due to their morphology and the internal lipid membrane and, therefore, a crucial role of the GroES/GroEL complex in MetSV assembly was postulated, as was shown for PRD1 [8,15,16]. However, this could not be proved in the current study.

#### 4.2.4. Global Mechanisms in MetSV-Infected Cells and Their Potential Role in Cell Lysis

The TEM analysis showed no induction of lysis-related host structures, most likely because cells burst due to a more general systemic reaction. Membrane destabilization in combination with osmotic pressure would lead to bursting of host cells. To find evidence for this, the current dual-RNAseq dataset was searched for global mechanisms, which might regulate central infection processes e.g., cell lysis. Here, *MM_RS14840* showed an outstanding transcription in comparison to all the other ORFs, because it was the only one out of two host-derived ORFs within the clusters of interest, which were significant differentially transcribed at 30 min p.i. The *MM_RS14840*-encoded protein is carrying a potential EF-hand domain, which was described to mediate Ca^2+^ ion interaction [68,69]. There is a huge number of studies of calcium-binding proteins and their roles in various cellular processes in humans, animals, and plants [70,71]. One prominent eukaryotic example of an EF-hand domain protein is calmodulin, which has diverse functions in the regulation of calcium homeostasis or calcium-dependent signal cascades e.g., high level Ca^2+^ induced cell death via calcineurin (Ca^2+^/calmodulin-dependent serine/threonine phosphatase) in neurons [72]. Calcium-binding proteins, such as calcium sensors, were also found in prokaryotes, but have not been studied in comparable depth to date [73]; for example the recently characterized calcium sensor EfhP of *Pseudomonas aeruginosa* has an important role in infection processes, biofilm formation, oxidative stress, and calcium homeostasis [74]. The regulation of *MM_RS14840* a t30 min p.i. suggests a comparable role in Ca^2+^ signaling in the context of the early MetSV infection processes, rather than a role in lysis-related pathways.

In the group of significantly differentially transcribed genes, additional genes were found which, in contrast to *MM_RS14840*, are more likely to play a role in MetSV-mediated cell lysis. One example is *MM_RS01165*, which encodes a putative membrane-associated cysteine proteinase. Here, *MM_RS01165* was significantly transcribed more frequently in MetSV-infected *M. mazei* cells a 180 min p.i. Cysteine proteinases were described to play crucial roles in entrance and egress of eukaryotic parasites in humans; for example, the egress of the malaria pathogen *Plasmodium* sp. From infected erythrocytes was found to be inhibited by L-transepoxy-succinyl-leucylamido-(4-guanidino)butane (E64), a cysteine proteinase inhibitor [75]. Cysteine proteinases were discussed to be responsible for membrane destabilization. These enzymes were further described to be translated as inactive precursor proteins which were activated by post-translational modification [75,76]. Therefore, *MM_RS01165* could play an important role in *M. mazei* cell lysis during MetSV infection and could be an efficient target for induced cell lysis. Cysteine proteinases were also described to induce ROS formation, which would have an additional negative effect on host cell integrity [77,78]. The second example is *MM_RS15030*, which encodes a pirin family protein. Here, *MM_RS15030* was, in similar manner to the previously described cysteine proteinase, >15-fold more transcribed at 180 min p.i. Pirin family proteins were described to play a role in apoptosis in plants, in the stimulation of tumorigenesis in mammals, or under various stress conditions e.g., salt stress in *Synechocystis* sp. *PCC 6803*, and in changes in redox-homeostasis and oxidative stress (ROS) in *Streptomyces* sp. [79,80,81,82,83]. In context of the MetSV infection, pirin might be a sensor for cellular degradation processes leading to cell lysis or could have some effect on cell lysis on its own. Additionally, *MM_RS17670,* encoding a solitary HicB protein (HicB-solo), was transcribed in similar manner. Solitary HicB proteins were discussed to mediate toxic effects, but were not characterized so far; the HicB3 found in *Yersinia pestis* is one example [84,85]. A role of the HicB variant encoded by *MM_RS17670* in cell lysis can be postulated, but must be proven by additional biochemistry experiments. All these genes and their encoded proteins together or individually could have important functions for the virus-mediated host cell lysis of *M. mazei*.

## 5. Hypothetical Infection Model Summarizes Conclusions

**Figure 6:**
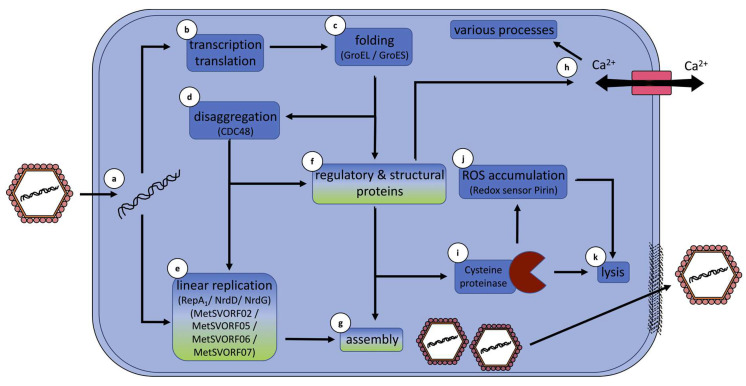
Hypothetical infection model summarizing general findings. Graphical summary of the involvement of host genes in combination with viral ORFs in MetSV infection processes. Host-encoded proteins and belonging processes are highlighted in blue; MetSV-derived proteins and belonging processes are highlighted in green. Gradient-filled boxes represent processes using viral and host proteins. Viral DNA is transferred into the host cell via an unknown process (**a**). *M. mazei* transcription–translation machinery is expressing viral ORFs (**b**). Viral proteins and protein complexes are folded and assembled under host chaperones e.g., GroEL/GroES (**c**). The MetSVORFs with a potential role in viral replication are held in solution via CDC48-mediated disaggregation (**d**). Linear replication is mediated by using hosts’ SSB RepA_1_ and dNTP production by the *nrdHDG* operon (**e**). Regulatory or structural viral proteins are used after folding and potential disaggregation to mediate viral particle formation, but these further influence cellular processes via calcium homeostasis (**f**–**h**). Induction of host-encoded proteins e.g., the membrane-associated cysteine proteinase (**i**), and the accumulation of ROS (**j**), could lead to cell lysis and virus particle release (**k**).

This study, the first dual-RNAseq of a virus infection of an archaeon, obtained a deeper knowledge of the MetSV infection cycle and generated a first infection model. Thereby, MetSV infection drastically alters host transcription and has a high impact on the host metabolism. After viral attachment to the host cell, viral DNA is transferred into the cell via an unknown process (Figure 6a). In the next step, host transcription/translation machinery is captured to express viral genes (Figure 6b). Resulting viral proteins could be folded via GroEL/GroES or similar processes (Figure 6c). Putative replication-dependent proteins, such as MetSVORF02 and MetSVORF05-MetSVORF7, are held in solution via CDC48-mediated disaggregation (Figure 6d). Linear genome replication is further performed under the influence of host-encoded SSB RepA_1_ and the dNTP production of the *nrdHDG* operon-encoded proteins (Figure 6e). Structural viral proteins could be used after folding and potential disaggregation to mediate viral particle formation. Virus assembly takes place in the cytoplasm of *M. mazei* without any apparent association with the cell membrane (Figure 6g). Potential regulatory proteins could further affect cellular processes, potentially via changes in calcium homeostasis (Figure 6f–h). Induction of the host-encoded membrane-associated cysteine proteinase (Figure 6i), as well as further discussed proteins and the potential accumulation of ROS (Figure 6j), could lead to cell disruption and virus particle release (Figure 6k).

## Data Availability

Read data can be found on NCBI database under BioProject accession number: PRJNA883050. R scripts for filtering, data analysis and visualization of raw read counts and tpm normalized counts of Dual-RNAseq data, as well as qRT-PCR result tables will be provided by request.

## Supplementary Materials

The following supporting information can be downloaded at: https://www.mdpi.com/article/10.3390/v14112585/s1. Figure S1: Ultrastructural analysis of MetSV infected *M. mazei* cells at 30 min p.i.; Table S1: List of used primers; Table S2: Final Dual-RNAseq dataset; Table S3: Significant subset of 285 genes; Table S4: PADLOC results of *M. mazei* genome.
