# Supplementary figures and images for: Dual-RNAseq Analysis Unravels Virus-Host Interactions of MetSV and Methanosarcina mazei
Source: Viruses. 2022 Nov 21;14(11):2585. doi: 10.3390/v14112585 (PMC9694453; doi:10.3390/v14112585)

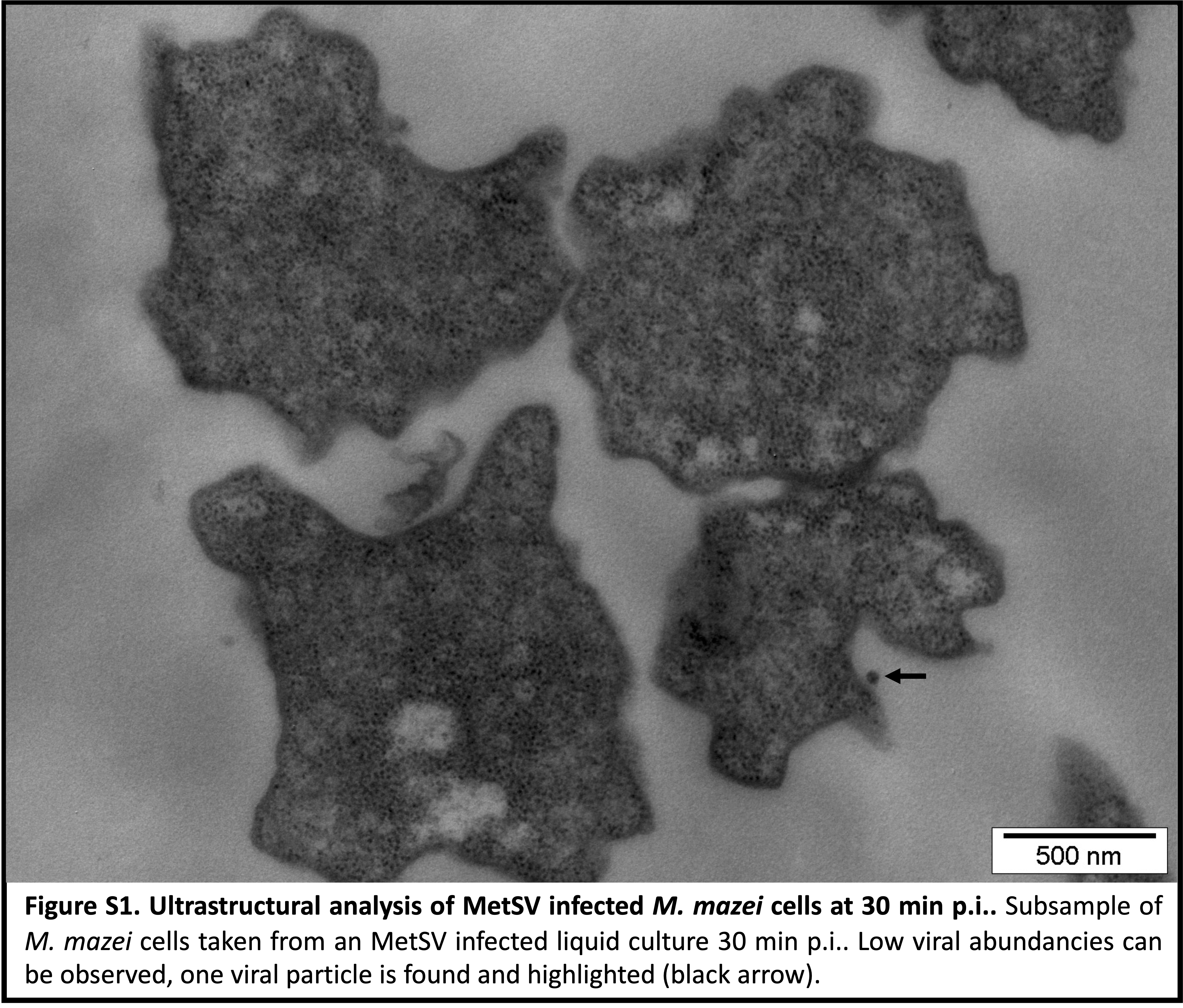

Supplement: Supplementary file 1 [file viruses-14-02585-s001.zip › Figure_S1.tiff]
